# Potential Effect of Giant Freshwater Prawn Shell Nano Chitosan in Inhibiting the Development of *Streptococcus mutans* and *Streptococcus sanguinis* Biofilm *In Vitro*

**DOI:** 10.1155/2023/8890750

**Published:** 2023-02-10

**Authors:** Tetiana Haniastuti, Tira Aisah Puspasari, Enggardini Rachma Hakim, Regina TC. Tandelilin

**Affiliations:** ^1^Oral Biology Department, Faculty of Dentistry, Universitas Gadjah Mada, Yogyakarta, Indonesia; ^2^Master of Dental Sciences Study Program, Faculty of Dentistry, Universitas Gadjah Mada, Yogyakarta, Indonesia; ^3^Oral Biology Department, Faculty of Dentistry, University of Muhammadiyah, Semarang, Indonesia

## Abstract

An oral biofilm comprises a variety of bacteria including *Streptococcus mutans* and *Streptococcus sanguinis* that cause human infections, such as caries and periodontitis. Thus, biofilm management plays an important part in the prevention and treatment of oral diseases. Nano chitosan is a bioactive material that has antimicrobial activities. This *in vitro* study aimed to evaluate the effect of nano chitosan synthesized from giant freshwater prawn shells (PSNC) on *S. mutans* and *S. sanguinis* biofilm development. PSNC was prepared from the extracted chitosan of giant freshwater prawn (*Macrobrachium rosenbergii*) shells using the ionic gelation method. The effect of PSNC on *S. mutans* ATCC 25175 and *S. sanguinis* ATCC10556 biofilm formation was evaluated using the crystal violet assay. Both bacteria were inoculated in the presence of various concentrations (5, 2.5, and 1.25 mg/ml) of PSNC for 24 h and 48 h. Confocal laser scanning microscopy (CLSM) and scanning electron microscopy were performed to visualize and study the biofilm architectural features. The biofilms were stained with the BacLight Bacterial Viability Kit prior to CLSM observation to monitor the viability of the biofilm. The results showed that PSNC exposure for 24 h and 48 h inhibited the formation of *S. mutans* and *S. sanguinis* biofilms. The biofilm formation inhibition percentage increased with an increase in the PSNC concentration (*p* < 0.05). The highest inhibitory activity was shown at 5 mg/ml PSNC (*p* < 0.05). Those findings were confirmed by the subsequent findings using the CLSM and SEM analyses. The biofilm architecture was strongly disrupted upon treatment with PSNC. After exposure to 5 mg/ml PSNC, the number of bacteria significantly decreased. The remaining bacteria were seen as individual cells, showing damaged cells. In conclusion, PSNC inhibits the development of *S. mutans* and *S. sanguinis* biofilm *in vitro*, indicating the potential of PSNC in clinical application for oral bacterial infection, prevention, and treatment.

## 1. Introduction

Oral biofilm, termed dental plaque, has been described as a structured microbial community that is attached to tooth surfaces and embedded in an extracellular polymeric matrix (EPS). The matrix of the biofilm protects the bacterial communities and makes them withstand harsh conditions and resist antimicrobial treatments [1]. The oral biofilm is composed of various microbial communities with approximately 700 distinct microbial species that interact with each other. This interaction can either positively or negatively affect the growth of the biofilm [2].

The accumulation of bacteria on the surfaces of the teeth and the gingival sulcus is considered the primary cause of dental caries, gingivitis, and periodontitis [3]. Dental caries is a biofilm-associated infectious disease fostered by biofilm dysbiosis that causes permanent destruction of the hard tissue of the tooth [4]. Dental caries is strongly associated with frequent exposure to dietary fermentable carbohydrates, leading to the accumulation of acid-producing and acid-resistant microorganisms on the surface of the teeth, thus creating an acidic pH environment. The overgrowth of acid-producing bacteria within biofilms leads to the formation of cariogenic biofilms that trigger caries [5]. The destruction of mineralized tooth tissues is the end result of the acidification of dental biofilm that progresses over time [6].

The sulcular and junctional epithelial cells are the first targets of the bacteria accumulating in the gingival sulcus. The cells react to the bacteria by altering cellular signaling, resulting in changes in cellular behavior, such as protease and cytokine production, cell proliferation, and cell migration [7]. A study by Currò et al. [8] showed that transglutaminase (TG) gene expressions in gingival tissues were altered in response to chronic injury. Transglutaminases are enzymes that contribute to determining cell shape by cross-linking proteins involved in cell adhesion and extracellular matrix stabilization. The study demonstrated a significantly downregulated level of TG1 and TG3 in gingival tissues of chronic periodontitis patients. The disintegration of the gingival epithelium makes it vulnerable to further bacterial invasion.

Furthermore, periodontitis is closely associated with various systemic diseases, including diabetes and cardiovascular diseases (CVDs) [9]. Periodontitis, through its chronic pathogenic biofilm burden, exerts a continuous negative stimulus on local host responses to secret high-sensitivity C-reactive protein (hs-CRP), nitric oxide, and various inflammatory mediators through mechanisms that are locally regulated by microRNAs (miRNAs).

Recent research has revealed that miRNAs have a role in a number of epigenetic processes connected to cardiovascular disease (CVD), increased oxidative stress, and also periodontitis [10]. A previous study by Isola et al. [11] showed that certain GCF miRNAs were considerably higher in individuals with periodontitis (miRNA-7 and -21), CVD (miRNA-7, -21, and -200), and individuals with periodontitis and CVD (miRNA-21).

*S. mutans* and *S. sanguinis* are important members of dental plaque and influence each other during oral biofilm formation [2]. *Streptococcus sanguinis* is a novel colonizer that aids the subsequent attachment of organisms and plays a significant role in oral biofilm development. Studies showed that *S. sanguinis* is one of the most abundant species in early dental biofilms. Although this bacterium is not known to be directly involved in oral disease, it is frequently implicated in infective endocarditis [12].

Normally, *S. mutans* lives as a regular member of the mature dental biofilm community; however, under certain circumstances, this bacterium becomes dominant and causes dental caries. *Streptococcus mutans* plays a significant role in tooth demineralization due to its ability to adhere to the tooth surfaces, generate acid, and also resist acid [13]. In addition, the bacteria have the capability to synthesize extracellular polysaccharides such as glucans or fructans via extracellular enzymes, namely, glucosyltransferase and fructosyltransferase [14].

Prior studies showed the antagonistic interaction between *S. mutans* and *S. sanguinis* at the ecological level [15–17]. However, recent findings prove otherwise. A study by AlEraky et al. [18] showed that *S. sanguinis* has been identified in patients with high levels of caries. Another study by Meriç et al. [19] reported similar frequencies of *S. mutans* and *S. sanguinis* in groups of caries- and caries-free subjects. In addition, a clinical study has shown that the interaction of *S. mutans* with *S. sanguinis* is an essential factor in caries status in children, suggesting that the relative levels of these two bacteria in the oral cavity play a significant role in the development of caries [20].

Oral biofilms are a major cause of oral infectious diseases; thus, biofilm management plays an essential role in the prevention and treatment of the diseases [4]. Considering that a number of studies have shown that immune response modulators such as tacrolimus are effective in the treatment of oral chronic inflammatory diseases (e.g., oral lichen planus and periodontitis), their use has attracted a great deal of interest [21–23]. *In vivo* study by Guimarães et al. [23] revealed that tacrolimus treatment in periodontitis-induced rats showed less bone loss associated with periodontitis, through a mechanism involving IL-1*β*, TNF-*α*, and IL-6. However, a study by Nivethitha et al. [24] reported a severe case of generalized gingival overgrowth caused by tacrolimus-induced treatment following a renal transplant.

Over the last few years, many chemical substances have been reported to have effects on bacterial cell adhesion. Some, such as chlorhexidine [25], delmopinol [26], and triclosan [27, 28] have shown potent inhibitory effects on biofilm development and maturation. Nevertheless, they exhibit several side effects such as tooth and tongue staining, taste alteration, and increased supragingival calculus formation [29]. Therefore, an attempt to explore new materials with minimal side effects to inhibit biofilm formation is necessary.

Chitosan is a natural polysaccharide derived from chitin, composed of 2-amino-2-deoxy-D-glycopyranose and 2-acetamide-2-desoxy-D-glycopyranose units linked by -1,4 glycosidic bonds. Chitosan is obtained by the deacetylation of chitin which is particularly abundant in shrimp shells, crab shells, crayfish, and krill shells [30]. Kumari et al. [31] reported that shrimp shells were found to be the best choice for chitosan production. All the physicochemical properties, such as the degree of deacetylation value, average molecular weight, and solubility data, support this. In addition, the XRD and FTIR patterns of shrimp chitosan were very similar to those of commercial chitosan.

Chitosan is used in biomedical applications due to its high biocompatibility and antimicrobial properties [32]. Several studies have shown that chitosan has antibacterial and antiplaque effects as well as antiadhesive properties against *S. mutans* and other *Streptococci* [33, 34]. A study by Costa et al. [34] revealed that chitosan was capable of inhibiting *S. mutans* adhesion and biofilm formation. Another study by Aliasghari et al. [33] demonstrated that chitosan and chitosan nanoparticles at a concentration of 5 mg/ml reduced *S. mutans* biofilm formation by up to 92.5% and 93.4%, respectively.

Chitosan nanoparticles are bioactive and environmentally friendly materials with unique physicochemical properties. Nano chitosan is a nanoparticle of a chitosan derivative product that has a smaller size than chitosan (10–1,000 nm) [35]. Smaller nano chitosan particle size can increase the solubility and penetration ability of the molecules into the biofilm [33, 36].

The accumulation of oral biofilm is one of the main causes of oral diseases that may lead to systemic diseases; thus, agents with antibiofilm properties are required for preventing the diseases. The search for new antibiofilm agents continues due to the lack of truly effective treatment options despite all the currently available products. Since bacteria exist in multispecies biofilms in the oral cavity, this *in vitro* research attempted to simulate the microecology of an oral Streptococci biofilm by cultivating *S. mutans* and *S. sanguinis* together. The purpose of the present study was to evaluate the effect of giant freshwater prawn shell nano chitosan (PSNC) on *S. mutans* and *S. sanguinis* biofilm development *in vitro*.

## 2. Materials and Methods

### 2.1. Extraction of Chitosan

Chitosan was extracted from freshwater prawn (*Macrobrachium rosenbergii*) shells through three major steps, namely, deproteination, demineralization, and deacetylation processes. In brief, the shells of the giant freshwater prawn were washed thoroughly with water to remove any adhering dirt and then dried in an oven at a temperature of 45°C for 3 days. The dry samples were pulverized and treated with 4% NaOH (Merck, Germany) for 60 minutes at a temperature of 80°C (deproteination). After the protein was depleted, the sample was subsequently washed thoroughly with distilled water and then dried at room temperature for 24 hours. The deproteinized sample was treated with 1 M HCl (Merck, Germany) for 3 hours to yield chitin (demineralization). The sample was washed thoroughly with distilled water to remove the excess HCl present in the chitin. Afterward, the demineralized sample was immersed in a 4% NaOCl solution. The deacetylation process was carried out by treating the sample with 50% NaOH for 3 hours at a temperature of 100°C. After washing thoroughly with distilled water, the sample was subsequently dried for 24 hours to obtain chitosan isolates.

### 2.2. Preparation of Giant Freshwater Prawn Shell Nano Chitosan (PSNC)

Nano chitosan was created by the ionic gelation method. Chitosan was dissolved in 1% acetic acid, and 1% tripolyphosphate (TPP) (Xilong AR, China) was then added drop by drop while stirring vigorously until it reached a ratio of chitosan to TPP = 2 : 1. The PSNC solution was dried using a spray dryer at a temperature of 120°C.

PSNC was dissolved in acetic acid (Merck, Germany) and kept under magnetic stirring overnight to completely dissolve the particles. The pH was adjusted with NaOH to a final value of 6.0. To obtain the desired volume, distilled water was added.

### 2.3. Microorganisms and Inoculum Preparation

*S. mutans* ATCC 25175 and *S. sanguinis* ATCC10556 were provided by the Integrated Research Laboratory, Faculty of Dentistry, Universitas Gadjah Mada, Indonesia. A single colony of *S. mutans* or *S. sanguinis* was cultured in BHI broth medium at 37°C overnight. The turbidity of the bacterial suspension was then adjusted to 0.5 McFarland (1.5 × 10^8^ CFU/mL).

### 2.4. Biofilm Formation Inhibition Assay

The effect of PSNC on *S. mutans* ATCC 25175 and *S. sanguinis* ATCC10556 biofilm formation was evaluated using a crystal violet assay. A total of 50 *μ*l BHI supplemented with 2% sucrose, 5 *μ*l of *S. mutans*, 5 *μ*l of *S. sanguinis*, and 40 *μ*l of various concentrations of PSNC (final concentration of PSNC was 5, 2.5, and 1.25 mg/ml), were transferred into a 96-well polystyrene microtiter plate (Iwaki, Japan). The plate was incubated at 37°C under anaerobic conditions for 24 h and 48 h. Phosphate-buffered saline (PBS) solutions and 0.2% chlorhexidine (CHX) were used as negative and positive controls, respectively.

After rinsing with PBS twice, the biofilms attached to the bottom of the microplates were stained with 125 *μ*l of 0.1% crystal violet for 15 min and washed with PBS to remove the residual dye. The bounded crystal violet was subsequently released with 200 *μ*l 96% ethanol. The absorbance of the released crystal violet in ethanol was recorded at OD540 nm by a spectrophotometer (Thermo Scientific, USA). The experiments were performed in quadruplicate. The inhibition percentage was calculated using the following formula [37]:

(1) $$\begin{array}{c} The\;percentage\;of\;biofilm\;inhibition=\frac{OD\;growth\;control-OD\;sample}{OD\;growth\;control}\times 100\text{.} \end{array}$$

### 2.5. Confocal Laser Scanning Microscopy (CLSM) Assay

Standard 24-microwell plates with sterilized glass coverslips were filled with the bacteria (*S. mutans* and *S. sanguinis*) in BHI media containing 2% sucrose and various concentrations of PSNC (the final concentrations of PSNC were 5, 2.5, and 1.25 mg/ml), as well as 2% Chx and PBS (controls). The plates were then incubated under anaerobic conditions for 24 h and 48 h at 37°C.

After the incubation period, the glass coverslips were rinsed with 0.15 M PBS (pH 7.0) and stained with the BacLight Bacterial Viability Kit (Thermofisher, USA) according to the manufacturer's instructions. The staining aimed to observe the viability of bacterial populations. The green stain indicated live cells, while the red stain indicated dead or dying cells. The stained biofilms were then observed with CLSM (Zeiss-Leica, Germany). The assay was performed in triplicate.

### 2.6. Biofilms Structure Observation by Scanning Electron Microscope (SEM)

Sterilized round glass coverslips were placed in 24 microwell plates and immersed with *S. mutans* and *S. sanguinis* in BHI media containing 2% sucrose, various concentrations of PSNC (the final concentrations of PSNC were 5 and 2.5 mg/ml) as well as PBS (control). The plates were incubated under anaerobic conditions for 24 h and 48 h at 37°C. After the incubation period, the coverslips were rinsed with PBS to remove unattached cells and fixed with 2.5% glutaraldehyde (Sigma-Aldrich, St Louis, MO, USA) overnight at 4°C. The coverslips were washed twice with PBS, subsequently dehydrated in a series of 50–100% ethanol solutions, and dried using a *vacuum system* (Buehler Cast n'Vac 1000, USA). The samples were sputter-coated with gold (JEOL 780174712) in an auto-fine coater (JEOL JEC-3000FC, USA) and then observed using a JSM-6510LA SEM. The assay was performed in triplicate.

### 2.7. Statistical Analysis

The biofilm formation inhibition assay was independently repeated at least four times. Data were expressed as the mean ± standard deviation (SD) from a representative experiment. A one-way analysis of variance (ANOVA) followed by a post hoc LSD test was performed to determine the significance of the groups. Statistical analysis was performed using the SPSS software, version 16.0. A *p* value less than 0.05 was considered significant.

## 3. Result

### 3.1. Biofilm Formation Inhibition

**Figure 1:**
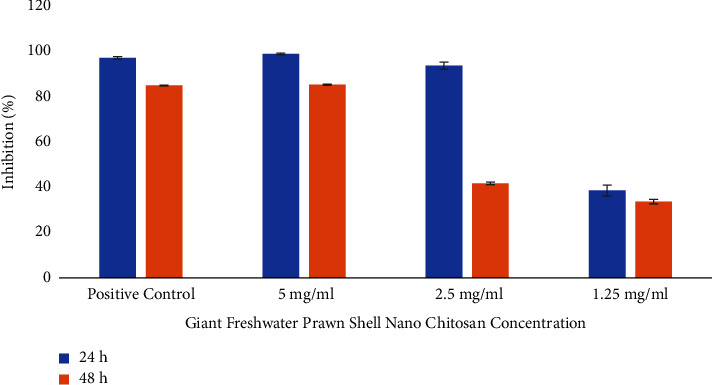
The effects of various concentrations of PSNC on biofilm formation. The biofilm formation inhibition percentage increased with an increase in the concentration of PSNC. Data were calculated from four independent assays and reported as the average; the standard deviations are indicated.

The effect of PSNC on biofilm formation is shown in Figure 1. Experimental data show biofilm formation inhibition percentages of 98.92 ± 0.30, 93.69 ± 1.59, and 38.58 ± 2.40 when the bacteria were exposed to 5, 2.5, and 1.25 mg/ml PSNC for 24 h, respectively. However, the biofilm formation inhibition percentage of 0.2% Chx (positive control) was 97.15 ± 0.55.

After being exposed to PSNC at concentrations of 5, 2.5, and 1.25 mg/ml for 48 h, the experimental data of biofilm inhibition percentages were 85.30 ± 0.26, 41.69 ± 0.57, and 33.66 ± 0.99, respectively. The results show that the biofilm formation inhibition percentage of 0.2% Chx was 84.94 ± 0.16 (Figure 1).

The results revealed that PSNC exposure for 24 h and 48 h inhibited the formation of *S. mutans* and *S. sanguinis* biofilms. The biofilm formation inhibition percentage increased with an increase in the concentration of PSNC (*p* < 0.05). Furthermore, 5 mg/ml PSNC showed the highest inhibitory activity among the other concentrations of PSNC (*p* < 0.05). LSD showed no significant differences (*p* > 0.05) between 5 mg/ml PSNC and 0.2% Chx at 24 h and 48 h exposure, indicating that both materials have the same effectiveness in inhibiting biofilm formation.

### 3.2. Confocal Laser Scanning Microscopy Observation

**Figure 2:**
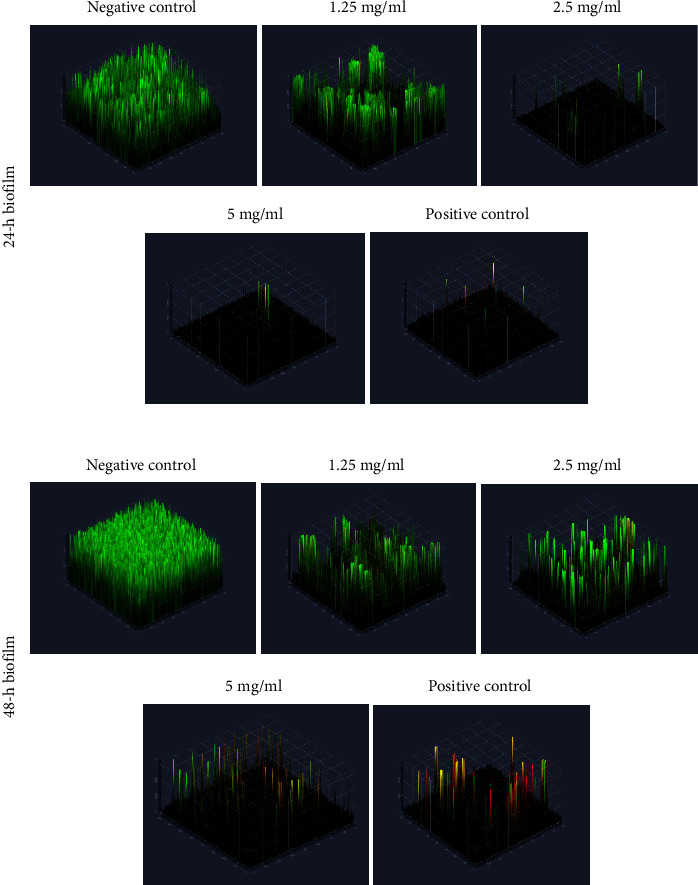
Representative 3D CLSM images of 24 h and 48 h *S. mutans* and *S. sanguinis* biofilms after exposure with varying concentrations of PSNC. The biofilm masses decrease as the concentrations of nano chitosan increase.

Based on the 3D CLSM images (Figure 2), 24 h *S*. *mutans* and *S*. *sanguinis* biofilms of the negative control group appear thick and densely distributed, most of which are stained green, indicating live cells. The CLSM images revealed substantial disintegration of the biofilm structure and decreased surface coverage after being treated with 1.25 mg/ml PSNC for 24 h. The biofilm appeared in clusters, and some blank areas on the surface were observed. The disruption of the biofilm was obviously increased as the concentration of PSNC increased. The bacteria were scattered and did not form biofilm in the sample exposed to 2.5 mg/ml and 5 mg/ml PSNC as well as 0.2% Chx (positive control). The number of bacteria was reduced significantly; the remaining bacteria were mostly stained red, indicating that they were dead or dying.

In comparison to the 24 h biofilm, the CLSM images of the 48 h of *S. mutans* and *S. sanguinis* biofilms of the negative control group appear thicker and denser. The biofilm showed green fluorescence, indicating active living cells. The formation of the biofilm was disrupted after the bacteria were exposed to PSNC. The CLSM images of samples treated with 1.25 mg/ml and 2.5 mg/ml PSNC for 48 h displayed thinner biofilm-containing cell clusters and decreased surface coverage. The disruption of the biofilm was most obvious at 5 mg/ml PSNC exposure. The cell clusters were scattered, and most bacteria were stained red, indicating dead or damaged cells. CLSM results from the group exposed to PSNC 5 mg/ml gave a similar picture to the positive control, indicating the same effectiveness in inhibiting biofilm formation (Figure 2).

### 3.3. Scanning Electron Microscope Observation

**Figure 3:**
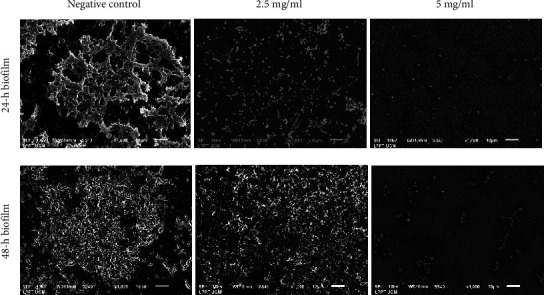
Representative SEM images showing reduction of *S. mutans* and *S. sanguinis* biofilm masses after exposure with varying concentrations of PSNC for 24 and 48 h. In untreated control groups, the biofilm is clearly visible with the bacteria embedded in EPS. The biofilm masses become less after being exposed to PSNC for 24 and 48 h.

SEM images of *S. mutans* and *S. sanguinis* biofilms are shown in Figure 3. In the control group, the 24 h biofilm covering the coverslips showed a typical pattern of biofilm with dense bacterial colonies. The bacteria overlapped and gathered in clusters and appear to be embedded in EPS. After 24 h of 2.5 mg/ml PSNC exposure, the number of biofilm cells that adhered to the glass coverslips diminished, and the cells showed a scattered distribution. The bacteria were arranged in the form of aggregates or as individualized cells. After being exposed to 5 mg/ml PSNC, the number of bacteria significantly decreases, and most of them are seen as individual cells.

Scanning electron micrographs of 48 h biofilm (Figure 3) appeared thicker and sheathed in a thicker matrix of biofilm than 24 h biofilm. In comparison to the negative control, after 48 h of 2.5 mg/ml PSNC exposure, the number of biofilm cells adhered to glass coverslips decreased, and the biofilm matrix was partially disrupted. The biofilm diminished after being exposed to 5 mg/ml PSNC. The remaining bacteria are arranged in the form of aggregates or simply as individualized cells.

## 4. Discussion

The results of the present study evidenced that PSNC inhibited *S. mutans* and *S. sanguinis* biofilm formation *in vitro*. Biofilms are complex microbial communities characterized by cells adhered to substrate surfaces, interfaces, or each other, embedded in cell-generated extracellular polymeric matrices (glycolipids, proteins, glycoproteins, and extracellular DNA) that protect the microbial community [1, 38]. Microbial behavior within biofilms differs significantly in terms of growth rate and gene transcription from the behavior of the same organisms studied under planktonic conditions. The bacteria residing within biofilms are more resistant to antibiotics than planktonic bacteria; thus, they play an important role in the development of chronic oral infections, including caries and periodontal diseases [39].

Caries and periodontal diseases are both complex in nature and share a variety of contributing factors that either directly or indirectly link them. Deep pockets of severe chronic periodontitis exhibit low oxygen tension, which may promote the growth of microaerophilic species like *S. mutans*. Previous studies revealed that untreated periodontitis patients have significant *S. mutans* recovery rates from saliva and subgingival plaque [40].

Bacteria interact with cells in oral tissues and trigger inflammatory responses that lead to tissue destruction. Evidence showed downregulated transglutaminase gene expression (TG1 and TG3) in gingival tissues of chronic periodontitis patients. This indicates a disruption of the structural integrity of the gingival epithelium, making the gingival epithelium susceptible to bacterial invasion [8].

Furthermore, bacteria negatively influence host responses to secret high-sensitivity C-reactive protein (hs-CRP), nitric oxide, and various inflammatory mediators through mechanisms that are locally regulated by miRNAs [10]. Recent research has revealed that miRNAs have a role in epigenetic processes associated with cardiovascular disease, increased oxidative stress, and periodontitis [9]. Several studies showed that periodontitis is significantly linked to cardiovascular diseases [10].

Chitosan is a natural linear polysaccharide produced from chitin by solid-state deacetylation under alkaline conditions or by enzymatic hydrolysis of chitin deacetylase. Some of chitosan's most notable properties in the medical context are its nontoxicity, biocompatibility, biodegradability, and immune-enhancing activities [36]. Previous studies have shown that nanosized chitosan has excellent activities such as antibacterial effects, drug delivery systems, gene and/or vaccine delivery systems, and antitumor effects [41–43]. In the present study, chitosan was extracted from giant freshwater prawn shells. The degree of deacetylation of chitosan obtained was 77.61%. The chitosan was further processed by ion gelation with TPP to produce PSNC. The average particle size of PSNC that formed was 432.9 nm with a polydispersity index = 0.288 and a molecular weight = 34.67 kDa. In addition, the zeta potential test result for PSNC was +51.2 (unpublished data).

The results of the crystal violet assays as well as CLSM and SEM analysis in the present study demonstrated that PSNC exposure for 24 and 48 h inhibited the development of *S. mutans* and *S. sanguinis* (dual species) biofilm. The antibiofilm effect on the dual-species biofilm increased as the concentration of PSNC increased. The present study revealed that the highest PSNC concentration examined (5 mg/ml) demonstrated the strongest antibiofilm activity since the biofilm architecture was strongly disrupted upon treatment. These results are consistent with those of previous studies [33, 44].

Aliasghari et al. [33] investigated the effects of chitosan and nano chitosan against single cariogenic bacteria (*S. mutans, S. salivarius, S. sanguinis,* and *S. sobrinus*) in planktonic and biofilm forms. The result of their study showed that chitosan and nano chitosan have bacteriostatic or bactericidal activities as well as antiadhesion effects against those bacteria. Furthermore, both materials reduce biofilm/plaque formation in vitro. Similar to our findings, they also revealed that by increasing the concentration of chitosan and nanochitosan, the antiadhesion activity on tested bacteria increased, and a 5 mg/mL concentration was found to be the most effective. Another study conducted by Costa et al. [44] found that chitosan-containing mouthwash was capable of hindering the biofilm formation and maturation of *S. mutans, L. acidophilus, E. faecium, C. albicans,* and *P. intermedia.*

A possible mechanism for PSNC's antibiofilm properties is due to its polycationic nature conferred by the functional amino group (NH3+) of the N-acetylglucosamine unit. The positive charges of PSNC are expected to electrostatically react with negatively charged biofilm components such as EPS, proteins, and DNA, resulting in inhibitory effects on bacterial biofilms [36, 45, 46]. Interaction between the polycationic nature of nano chitosan and the anionic sites of microbial cell membrane proteins results in the leakage of intracellular components, causing bacterial cell death [47]. In addition, the interaction may also result in bacterial cell flocculation, thus preventing the attachment of bacteria to the surface [48]. A study by Strand et al. [49] demonstrated that chitosan flocculated *Escherichia coli* suspensions.

Another possibility of the PSNC mechanism that inhibits biofilm formation is that PSNC interferes with protein synthesis, which affects the bacterial attachment. PSNC produced in this study had a small particle size (about 432.9 nm) and low molecular weight (34.67 kDa). The small particle size of PSNC may cause the molecules to easily penetrate the nucleus and bind to bacterial DNA [46]. In addition, PSNC with a low molecular weight more easily binds to the DNA of bacterial cells, thus disrupting the synthesis process of certain protein molecules, including proteins that support bacterial attachment [50]. Chávez de Paz et al. [51] demonstrated that chitosan nanoparticles prepared from low molecular weight chitosan induced >95% damage to *S. mutans* biofilms.

Due to the small size of PSNC particles (432.9 nm), PSNC may penetrate the bacterial cell wall and accumulate in the bacterial cell membrane, which in turn may interfere with bacterial cell metabolism [52]. In addition, electrostatic interactions between nano chitosan molecules and bacterial cell membranes may cause changes in cell membrane permeability. The alteration of the bacterial cell membrane may then affect their hydrophobicity which in turn prevents the adherence of the bacteria to the surface, thus inhibiting biofilm formation [53].

In conclusion, the results of the present study provide evidence that PSNC inhibits the development of *S. mutans* and *S. sanguinis* biofilm *in vitro*. The findings indicated the high potential of PSNC as antiplaque and anticaries agent, suggesting their potential application in dental biomaterials that might be integrated into oral health care products such as mouthwash and toothpaste.

## Data Availability

The data supporting the results of this study are included in the article.
